# The Annual Trip to the Ice-rink: A Seasonal Cause of Wrist Trauma in Irish Hospitals

**DOI:** 10.7759/cureus.6757

**Published:** 2020-01-23

**Authors:** Martin Davey, Matt Davey, Marc C Grant-Freemantle, Sean Flynn, Gavin McHugh

**Affiliations:** 1 Trauma and Orthopaedics, Royal College of Surgeons, Dublin, IRL; 2 Plastic, Aesthetic, and Reconstructive Surgery, Galway University Hospitals, Galway, IRL; 3 Trauma and Orthopaedics, Beaumont Hospital, Dublin, IRL

**Keywords:** trauma, orthopaedics, ice, skating, ireland, wrist, fracture

## Abstract

Fractures of the distal radius are a common orthopaedic presentation in Irish emergency departments. As a nation, Irish people tend to ice-skate seasonally with a peak of interest seen during the Winter months in temporary ice-rinks. This case series describes winter ice-skating as a significant cause of wrist fractures in the younger patient, including five cases of distal radius fractures, four of which ultimately required internal fixation, under general anaesthesia, over a single weekend in the month of December. Despite all five patients being amateur ice-skaters, all denied ever having taken ice-skating lessons. This demonstrates the dangers of wrist trauma in the inexperienced or beginner ice-skaters on temporary ice-rinks; the seasonal morbidity suffered as a result.

## Introduction

Fractures of the distal radius are common presentations in the western world, accounting for over 16% of orthopaedic trauma presentations in emergency departments [1]. Nearly two-thirds of all distal radius fractures are reported to occur following low-velocity trauma in the elderly, osteoporotic patient. Such wrist fractures are reported to correlate with significant morbidity [2]. However, in younger patients, sporting injuries are reported to the leading cause of distal radius fractures [3]. 

Due to Ireland’s cool temperate oceanic climate, participation in ice-skating is typically only facilitated using non-natural ice-skating rinks. Therefore, peaks of interest in ice-skating are seen in Ireland during the Christmas period with seasonal ranks opening on a temporary basis in December nationwide. Despite the significant morbidity associated with this activity, many amateur Irish ice-skaters elect to ice-skate without having undergone lessons leading to a significant increase in ice-skating related injuries presenting to Irish emergency departments [4]. This case series demonstrates the dangers of wrist trauma associated with seasonal ice-skating and the morbidity suffered as a result.

## Case presentation

Case 1

**Figure 1:**
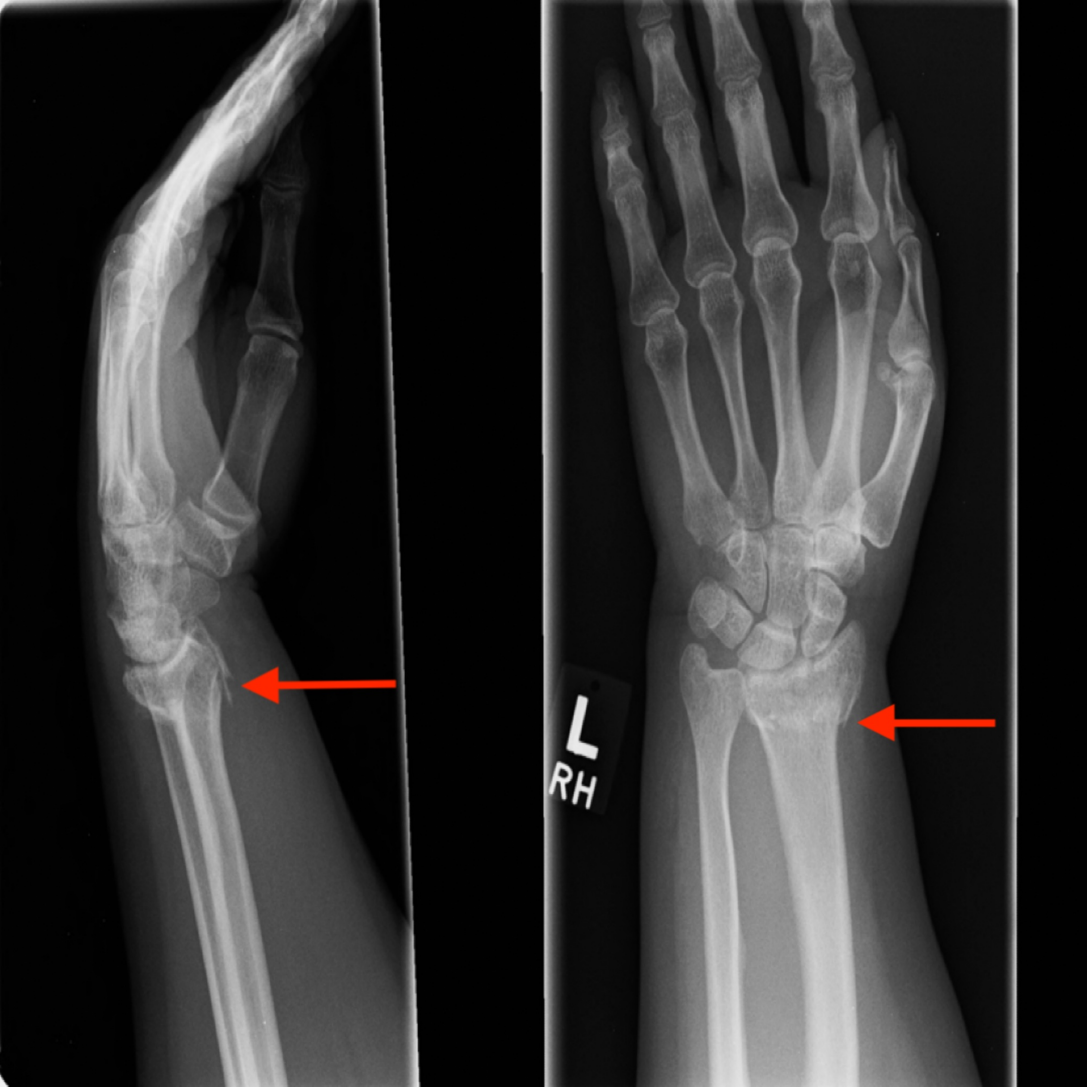
Extra-articular distal radius fracture discussed in Case 1

**Figure 2:**
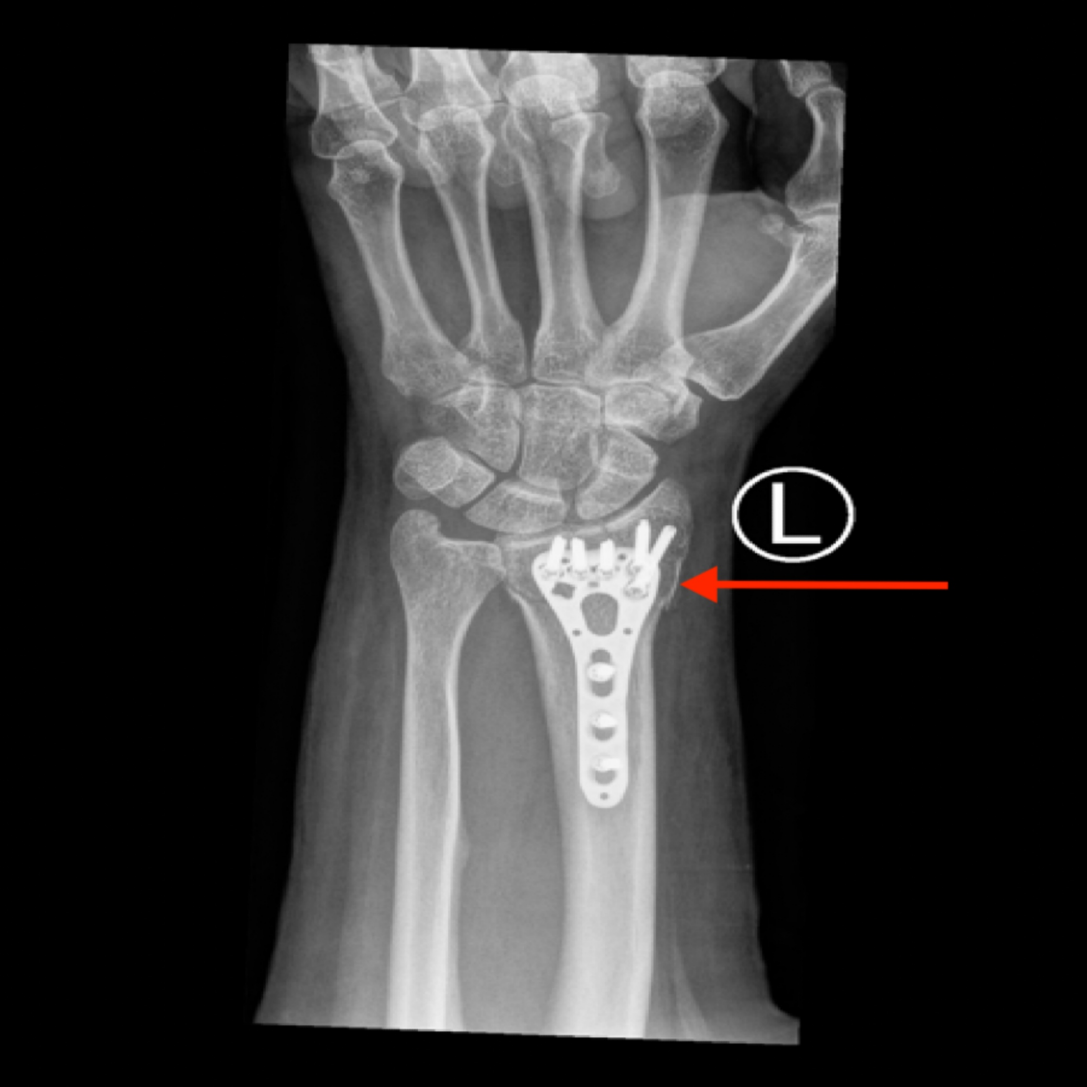
Open reduction and internal fixture of the distal radius fracture, of the patient discussed in Case 1, using a Variable Angle LCP Two-column Volar Distal Radius Plate LCP: Locking compression plate.

A 40-year-old right-hand-dominant lady presented to the emergency department following a fall on an outstretched hand (FOOSH) injury whilst ice-skating. She suffered an extra-articular distal radius fracture with dorsal comminution and angulation to her non-dominant left hand (Figure 1). Following the reduction in the emergency department, she was treated with open reduction and internal fixation using a Variable Angle LCP Two-Column Volar Distal Radius Plate 2.4 (DePuy Synthes Comp, IND, USA) (Figure 2).

Case 2

A 53-year-old right-hand-dominant lady presented to the emergency department following a fall backwards whilst holding hands with her friend while ice-skating. She suffered an extra-articular distal radius fracture with dorsal comminution and angulation to her dominant right hand; this had been the contra-lateral hand to that held whilst ice-skating. Following the reduction in the emergency department, she was treated with open reduction and internal fixation using an Angle Stable Distal Radial Plate System (Marquardt Group, Rietheim-Weilheim, Germany).

Case 3

A 36-year-old left-hand-dominant female teacher presented to the emergency department following a fall during her first-time ice-skating. She suffered an intra-articular distal radius fracture with dorsal angulation and severe comminution to her dominant left hand. She underwent open reduction and internal fixation using a Variable Angle LCP Two-Column Volar Distal Radius Plate 2.4; she remained off work during the holidays.

Case 4

A 41-year-old right-hand-dominant lady presented to the emergency department after a FOOSH injury whilst ice-skating. She suffered an intra-articular left distal radius fracture involving a significant portion of the radial styloid. She underwent closed reduction using two 1.6 mm Krischner wires (k-wires). She was neurovascularly intact post-operatively. Her k-wires were removed in the outpatient department at six-weeks with satisfactory fixation.

Case 5

A 37-year-old right-hand-dominant lady presented to the emergency department after falling backwards onto an out-stretched left hand whilst ice-skating. This resulted in an extra-articular left distal radius fracture with dorsal comminution. She was neurovascularly intact. This lady was treated conservatively using a molded cast and followed up in the outpatient fracture clinic thereafter.

## Discussion

Distal radius fractures are reported to occur more commonly in elderly, osteoporotic patients [4]. Our case series discusses the intermittent, annual trips to an ice-skating rink as a significant cause of morbidity for the young, active patient. As all five distal radius fractures presented over one weekend in December; this sparked curiosity for the authors. Ice-skating is a seasonal leisure activity in Ireland that accounts for significant trauma, particularly in the month of December [4]. 

As a nation, ice-skating remains a novelty to the Irish population, which is commonly enjoyed during the Christmas period [4]. Ice-skating injuries, although serious in nature, are thought to represent less than 1% of emergency department referrals during this time period [5]. Due to the sporadic nature of our engagement with the activity, few people elect to receive full ice-skating lessons prior to skating in a full-sized ice-rink [6]. Williamson et al. postulated that of those who sustain injuries whilst ice-skating, 75% will be beginners and 92% will never have received formal tuition or lessons [7]. Similarly, Matsumoto et al. reported that of those who suffer distal radius fractures during winter sporting and leisure activities, nearly 95% will never have had formal professional instruction [8]. 

Significant trauma can occur as a result of ice-skating and serious injuries (including distal radius fractures) related to this activity tend to occur in beginner ice-skaters [9]. Despite the majority of ice-skating injuries occurring in inexperienced or beginner ice-skating, over 40% of advanced ice-skaters will suffer a severe injury on the ice during their lifetime [10]. Of the trauma which occurs, it has been shown that upper limb trauma remains the commonest injury presenting to emergency departments following ice-skating accidents, with studies reporting that distal radius fractures may account for 45%-82% of such presentations [4,11].

## Conclusions

This case series demonstrates the risk distal radius fractures in light of our desire to enjoy the hazardous activity of ice-skating on the annual trip to the temporary ice-rink. Furthermore, acknowledgement must be given to the fact that ice-skating lessons may need to be more widely available to newcomers and amateur ice-skaters. This, alongside significant public education, may play a role in the future to reduce the burden on our emergency departments.

## References

[1] Chung KC, Spilson SV (2001). The frequency and epidemiology of hand and forearm fractures in the United States. J Hand Surg.

[2] Niempoog S, Sukkarnkosol S, Boontanapibul K (2019). Prevalence of osteoporosis in patients with distal radius fracture from low-energy trauma. Malays Orthop J.

[3] Lawson GM, Hajducka C, McQueen MM (1995). Sports fractures of the distal radius — epidemiology and outcome. Injury.

[4] Clarke HJ, Ryan D, Cusack S (2006). The impact of a temporary ice-rink on an emergency department service. Eur J Emerg Med.

[5] Barr LV, Imam S, Owen PJ (2010). Skating on thin ice: a study of the injuries sustained at a temporary ice skating rink. Int Orthop.

[6] Brown MG (1989). Ice rink injuries: a new epidemic in Northern Ireland. Ulster Med J.

[7] Williamson DM, Lowdon IMR (1986). Ice-skating injuries. Injury.

[8] Matsumoto K, Sumi H, Sumi Y, Shimizu K (2004). Wrist fractures from snowboarding. Clin J Sport Med.

[9] Radford PJ, Williamson DM, Lowdon IM (1988). The risks of injury in public ice skating. Brit J Sports Med.

[10] Dubravčić-Šimunjak S, Kuipers H, Moran J, Šimunjak B, Pećina M (2006). Injuries in synchronized skating. Int J Sports Med.

[11] Kelsal NKR, Bowyer GW (2009). Injuries sustained at a temporary ice-skating rink: prospective study of the Winchester experience 2007-2008. Injury.

